# Teacher led school-based surveillance can allow accurate tracking of emerging infectious diseases - evidence from serial cross-sectional surveys of febrile respiratory illness during the H1N1 2009 influenza pandemic in Singapore

**DOI:** 10.1186/1471-2334-12-336

**Published:** 2012-12-04

**Authors:** Mark IC Chen, Chia Yin Chong

**Affiliations:** 1 National University of Singapore, 21 Lower Kent Ridge Road, Singapore 119077, Singapore; 2 Communicable Disease Centre, Tan Tock Seng Hospital, 11 Jalan Tan Tock Seng, Singapore 308433, Singapore; 3 Duke-NUS Graduate Medical School, 8 College Road, Singapore, 169857, Singapore; 4 Ministry of Defence, Gombak Drive, Singapore, 669645, Singapore; 5 Ministry of Health, College of Medicine Building, 16 College Road, Singapore, 169854, Singapore; 6 KK Women’s and Children’s Hospital, 100 Bukit Timah Road, Singapore, 229899, Singapore; 7 World Health Organization Collaborating Centre for Reference and Research on Influenza, 10 Wreckyn Street, North Melbourne, VIC, 3051, Australia; 8 National University Health Systems, 1E Kent Ridge Road, Singapore, 119228, Singapore; 9 Department of Clinical Epidemiology, Tan Tock Seng Hospital, 11 Jalan Tan Tock Seng, Singapore, 308433, Singapore

**Keywords:** Respiratory tract infections, Vaccination, Serology

## Abstract

**Background:**

Schools are important foci of influenza transmission and potential targets for surveillance and interventions. We compared several school-based influenza monitoring systems with clinic-based influenza-like illness (ILI) surveillance, and assessed the variation in illness rates between and within schools.

**Methods:**

During the initial wave of pandemic H1N1 (pdmH1N1) infections from June to Sept 2009 in Singapore, we collected data on nation-wide laboratory confirmed cases (Sch-LCC) and daily temperature monitoring (Sch-DTM), and teacher-led febrile respiratory illness reporting in 6 sentinel schools (Sch-FRI). Comparisons were made against age-stratified clinic-based influenza-like illness (ILI) data from 23 primary care clinics (GP-ILI) and proportions of ILI testing positive for pdmH1N1 (Lab-ILI) by computing the fraction of cumulative incidence occurring by epidemiological week 30 (when GP-ILI incidence peaked); and cumulative incidence rates between school-based indicators and sero-epidemiological pdmH1N1 incidence (estimated from changes in prevalence of A/California/7/2009 H1N1 hemagglutination inhibition titers ≥ 40 between pre-epidemic and post-epidemic sera). Variation in Sch-FRI rates in the 6 schools was also investigated through a Bayesian hierarchical model.

**Results:**

By week 30, for primary and secondary school children respectively, 63% and 79% of incidence for Sch-LCC had occurred, compared with 50% and 52% for GP-ILI data, and 48% and 53% for Sch-FRI. There were 1,187 notified cases and 7,588 episodes in the Sch-LCC and Sch-DTM systems; given school enrollment of 485,723 children, this represented 0.24 cases and 1.6 episodes per 100 children respectively. Mean Sch-FRI rate was 28.8 per 100 children (95% CI: 27.7 to 29.9) in the 6 schools. We estimate from serology that 41.8% (95% CI: 30.2% to 55.9%) of primary and 43.2% (95% CI: 28.2% to 60.8%) of secondary school-aged children were infected. Sch-FRI rates were similar across the 6 schools (23 to 34 episodes per 100 children), but there was widespread variation by classrooms; in the hierarchical model, omitting age and school effects was inconsequential but neglecting classroom level effects led to highly significant reductions in goodness of fit.

**Conclusions:**

Epidemic curves from Sch-FRI were comparable to GP-ILI data, and Sch-FRI detected substantially more infections than Sch-LCC and Sch-DTM. Variability in classroom attack rates suggests localized class-room transmission.

## Background

School-aged children have higher influenza infection rates than adults [1], and school outbreaks often have high attack rates, possibly due to the increased social interaction in schools, low immunity levels and higher levels of viral shedding [2,3]. Schools are important foci of influenza transmission [4,5], and growing evidence suggests school closures may reduce transmission during epidemics [6-8]. During the 2009 influenza A H1N1 pandemic, school outbreaks often preceded epidemics in the general community [7,9]. Schools are hence a potential target population for surveillance [10] and interventions for reducing transmission both within the school environment and the wider community [6].

Following its emergence in North America in early 2009, pandemic H1N1 (pdmH1N1) spread to Singapore with the first imported case diagnosed on 26 May 2009 [11]. The onset of community transmission in Singapore (mid-June 2009) coincided with the scheduled mid-year school holiday, with schools re-opening on 29 June 2009. During the initial wave of infections from mid-June to mid-September 2009 [12], several systems were in place to monitor the incidence of infection and to detect and intervene in outbreaks within schools in Singapore [13]. These included notifications of laboratory confirmed cases throughout the epidemic; a system of daily temperature monitoring for students (also used during the Severe Acute Respiratory Syndrome outbreak in 2003) [14]; and a novel teacher-led febrile respiratory illness (FRI) reporting system in 6 sentinel schools to track the epidemic’s progress as well as identify possible instances of localized transmission. However, it remains unclear how effective these school-based surveillance systems were, and if they should again be deployed in future epidemics.

In this study, we describe our observations from various school-based systems, and compare these against observations from clinic-based influenza-like illness (ILI) surveillance. We compare the epidemic curves, and estimate the incidence of febrile illness and infections detected by the different systems and in cross-sectional serological surveys. We also assess the variation in FRI rates by schools and classes within schools, and discuss the implications that this and our other findings may have for detection and control of influenza transmission in schools.

## Methods

### Study setting

This study was performed on primary and secondary school children in Singapore, typically aged 7 to 12 and 13 to 16 years respectively, from 12 June 2009 (epidemiological week 23), when pdmH1N1 infection was first confirmed in a school child, to 3 October 2009 (epidemiological week 39), when national acute respiratory illness rates returned to baseline levels [15].

### Overview of datasets analyzed

The study included several clinic-based indicators of influenza activity (see Table 1), analyzed by the relevant age strata (7 to 12, and 13 to 16 years for primary and secondary school-age children respectively):

– Influenza-like illness reporting from sentinel general practitioners (GP-ILI)

– Proportion of influenza-like illness samples from primary care clinics testing positive by PCR (Lab-ILI)

**Table 1:** Description of measures of influenza-related illness during initial epidemic of pdmH1N1

**Measure**	**Description**	**Period analyzed**	**No. at-risk/samples tested**		**No. of episodes/positive tests**	
			**Pri**	**Sec**	**Pri**	**Sec**
**GP-ILI**: No. of ILIs per GP doctor	Based on ILI reporting by network of 23 GPs, where numerator is number of ILI reports and denominator is number of GPs reporting per day (median 14/d, IQR: 10-15); ILI is defined here as a consult for acute respiratory illness with a temperature of 38°C and above	25 Jun 2009 to 10 Oct 2009 (weeks 25 to 40)^1^	NA	NA	440	279
**Lab-ILI**: Proportion of ILIs at GP clinics testing positive for pdmH1N1	Based on testing of ILI samples from a separate sentinel GP network by the National Public Health Laboratory, where numerator is number of samples positive for pdmH1N1 and denominator is number of samples tested; ILI is defined here as a consult for acute respiratory illness with a temperature of 38°C and above	21 Jun 2009 to 10 Oct 2009 (weeks 25 to 40)^2^	1,372	1,029	878	656
**Sch-LCC:** Lab confirmed cases of pdmH1N1	Notifications of laboratory confirmed cases of pdmH1N1 as compiled by the Ministry of Education	12 Jun 2009 to 7 Oct 2009 (weeks 23 to 40)	270,344^3^	215,379^3^	654	533
**Sch-DTM:** Daily tempe-rature moni-toring	Temperature taking of students conducted twice daily in all schools; counts of the number of students detected to have fever on twice daily temperature taking is presented, with fever defined as 37.9°C and above for children of ages 12 years and below and 37.6°C and above for children of ages 13 years and above	30 Jun 2009 to 24 Jul 2009 (weeks 26 to 29)^4^	270,344^3^	215,379^3^	4,656	2,932
**Sch-FRI**: Febrile respiratory illness reporting	3 primary and 3 secondary schools from different areas of Singapore were selected to report FRI episodes, with FRI defined as fever of ≥ 37.5°C accompanied by either cough or sore throat; data was collated by the class teacher-in-charge once every 2 weeks	29 Jun 2009 to 3 Oct 2009 (weeks 26 to 39)^5^	4,320	4,821	964	1,178
**Pre-SS** and **Post-SS**: Pre-epidemic and post-epidemic serological surveys	Age-stratified serological data based on the proportion with hemagglutination inhibition titers ≥ 40 to pdmH1N1 in post-epidemic samples (Post-SS) minus that in pre-epidemic samples (Pre-SS), adjusted for the sensitivity of detecting confirmed infections using cross-sectional hemagglutination inhibition titers ≥ 40 to pdmH1N1	Pre-SS: 1 Feb 2008 to 31 May 2009	381	321	3	34
		Post-SS: 1 Oct 2009 to 2 Jun 2010	124	96	41	39

1 Estimates for epidemiological week 25 were extrapolated from the ILI rate for the 3 days (25 June to 27 June 2009) where data was available.

2 Only data from these dates were extracted, though surveillance extends before and after these dates.

3 Based on school enrolment on 29 June 2009.

4 Data on 29 June 2009 discarded due to inaccuracies in reporting on first day of school.

5 Up to week 34 for all 6 schools, and up to week 39 for 2 primary and 2 secondary schools.

GP-ILI was used separately and in combination with Lab-ILI to provide an indication the epidemic’s progress against which the following school-based indicators could be compared:

– Notifications of laboratory confirmed cases of pdmH1N1 by school (Sch-LCC)

– Students with fever detected by a system of twice daily temperature monitoring in all schools (Sch-DTM)

– Self-reported febrile respiratory illness in 6 sentinel schools (Sch-FRI)

Finally, cross-sectional serological surveys were used to estimate the amount of pdmH1N1 infection in school-aged children. Details on each of the datasets and associated analyses follow.

### Clinic-based indicators of epidemic activity

A sentinel network of 23 GPs was set up to monitor pdmH1N1 influenza epidemic activity [16], where participating GPs submitted individual level data on the age, residency status and body temperature for patients seen at their clinic with ILI from 25 June 2009 to 10 October 2009. We extracted data for Singapore residents of primary and secondary school-age by epidemiological weeks (from weeks 25 to 40); ILI here was defined as fever of ≥ 38.0°C in the presence of acute onset respiratory symptoms (nasal congestion, cough and/or sore throat), and expressed as the number of ILI consults per GP per day.

In addition, we obtained from the National Public Health Laboratory of Singapore age-stratified weekly data on the number of samples submitted and the number testing positive by influenza PCR (Lab-ILI); these were from patients presenting with a similar case definition of ILI to sentinel primary care clinics submitted as part of the routine national influenza surveillance program.

Since similar case definitions for ILI were used in both datasets, the GP and laboratory data for corresponding time-points were also multiplied to provide a composite indicator, GP*Lab-ILI; this removes the contribution of non-pdmH1N1 causes of ILI to give an estimate of the number of ILI consults attributable to pdmH1N1 [17].

### School-based indicators of epidemic activity (Sch-LCC, Sch-DTM and Sch-FRI)

In Singapore, Influenza A pdmH1N1 was made legally notifiable to the Ministry of Health on 27 April 2009 [13], with medical practitioners required to notify all laboratory (predominantly RT-PCR) confirmed cases. The Sch-LCC dataset included anonymized individual level data on notified laboratory confirmed cases in school children from 12 June to 7 October 2009 provided to us by the Ministry of Education. Incidence of notified cases per 100 children was computed based on enrolment data from all 177 primary and 160 secondary schools in Singapore.

The Sch-DTM comprised daily counts of students per school identified with fever from a system of twice daily temperature monitoring. This was conducted nation-wide by teaching staff as part of efforts to detect and intervene in outbreaks [13] from 30 June to 24 July 2009, with fever defined as a temperature of ≥ 37.9°C for children aged 12 years and below, and a temperature of ≥ 37.6°C for children aged 13 years and above, the different age-specific cut-off points being based on a prior study [14]. This monitoring ceased after epidemiological week 29, when it had become clear that the pandemic strain was relatively mild [13].

Finally, the Sch-FRI data was from a project in three primary schools with 4,320 students and three secondary schools with 4,821 students in different parts of Singapore where our team had implemented febrile respiratory illness (FRI) monitoring with teacher-led reporting. FRI was defined as a reported fever of ≥ 37.5°C accompanied by cough and/or sore throat of acute onset. The class teacher-in-charge (trained by our research staff) collated these data, along with dates of illness onset and physician-certified sick leave, if any. The surveys were conducted fortnightly by teachers from 13 July until early October 2009, with successive surveys capturing new illness episodes. For FRI episodes with missing onset dates (724 out of 2,866 episodes, or 25%), we imputed a date uniformly over the period between the survey when the episode was reported and the preceding survey date (or the first day of school after the holidays for episodes reported in the first survey).

### Pre-epidemic and post-epidemic serological survey (Pre-SS and Post-SS)

We measured the seroprevalence of antibodies to influenza A/California/7/2009 in two sets of residual pediatric sera, collected before and after the pandemic. The laboratory methods and associated analysis are identical to those from our previous work [17-19], and are described further in the Additional file 1: Appendix along with additional details on the source of our samples.

### Statistical analysis

In addition to visually comparing the epidemic curves, we calculated the cumulative incidence up to epidemiological week 30 (when the epidemic peaked in the GP-ILI data) as a fraction of the cumulative incidence observed over the entire period for the same indicator. This was done for the GP-ILI and GP*Lab-ILI composite indicator, and also for the two school-based indicators (Sch-LCC and Sch-FRI) which were active up to epidemiological week 39. For Sch-FRI, we also derived an adjusted estimate (Sch-FRI-adj) where we attempted to remove possible contribution from non-pdmH1N1 causes by subtracting the FRI incidence in week 38; week 38 was used as a proxy for baseline FRI incidence since data for 5 of the 6 schools was still available for that week, and the proportion of ILI testing positive for pdmH1N1 had stabilized at about 20% by then. In addition, as an indication of the amount of pdmH1N1 infections detected, we computed the ratio of cumulative incidence rates up to week 39 for Sch-LCC and Sch-FRI-adj to the serologically estimated incidence of pdmH1N1 infections. Where appropriate, confidence intervals (CIs) were generated using binomial and Poisson distributions for proportions and cumulative incidence rates respectively.

To assess the variation in FRI incidence from the 6 schools, and between classrooms within the 6 schools, we quantified the relative importance of three sources of variability – school, age group, and classroom – on classroom FRI rates. We developed a hierarchical model in which the logit of the per capita probability of having a FRI was governed by school, age and classroom effects, with class grade used as proxy for age. Each in turn was assumed to be drawn from a mean zero normal distribution with non-informative prior distributions for the standard deviation. We then fitted more parsimonious models excluding one of these effects and assessed the concordance between the empirical and modeled (posterior predictive individual class level) FRI rates graphically. Relative goodness of fit was measured by the deviance information criterion (DIC), following the usual rule of thumb that models with DIC within 2 of each other have no effective difference in goodness of fit, while a DIC difference of more than 10 indicates substantial evidence in favor of the better (i.e. lower) scoring model. The model was fit within the Bayesian framework using JAGS v2.2.0, with four independent chains each run for 100 000 iterations following a burn-in period of 1 000 iterations, and every 10^th^ iteration retained for subsequent analysis (see Additional file 1: Appendix for mathematical formulation).

### Ethics review and informed consent

Teacher-led febrile respiratory illness reporting was reviewed by the ethics review board of the National University of Singapore, which approved the submission of anonymized data on febrile respiratory illness episodes in the participating schools without need for participant or parental consent.

## Results

### Primary care ILI and laboratory testing of ILI

**Figure 1:**
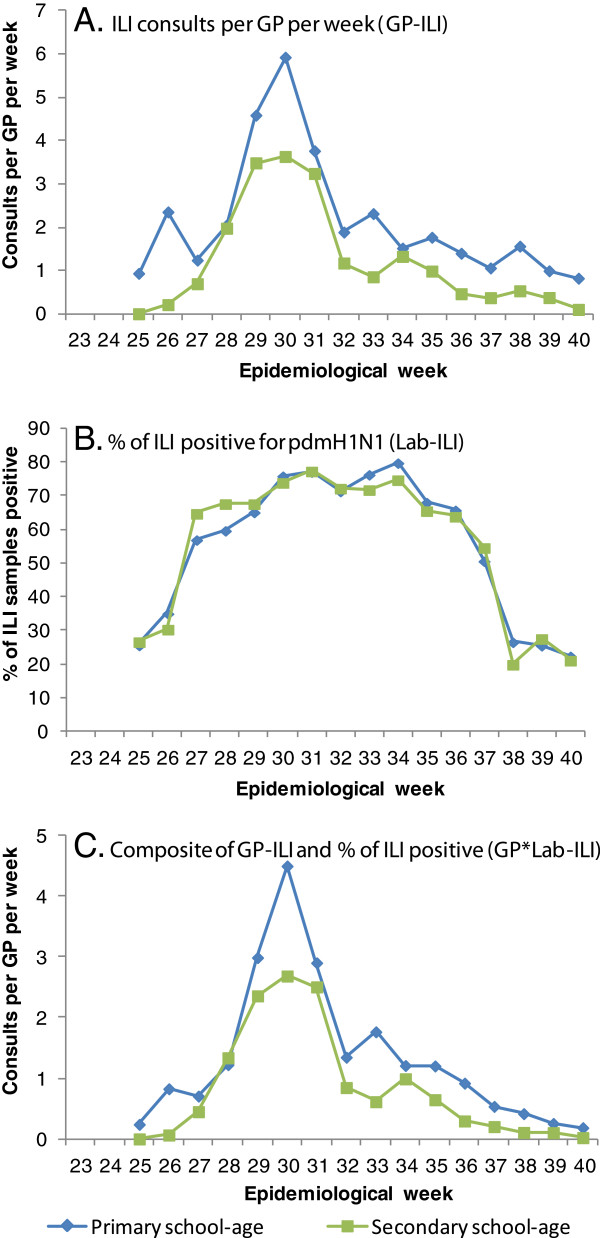
**Data derived from clinic-based indicators of epidemic activity from epidemiological week 25 to 40 (21 June 2009 to 10 October 2009). A**) GP-ILI data, expressed as ILI consults per GP per week. **B**) Lab-ILI data, expressed as weekly proportion of ILI samples positive for pdmH1N1. **C**) Composite indicator of GP*Lab-ILI (by epidemiological week) which gives the estimated ILI consults per GP per week attributable to pdmH1N1 influenza.

Figures 1A and B presents the epidemic curves for school-aged children as observed in the GP-ILI data, and the proportion of samples from ILI patients testing positive for pdmH1N1 respectively (Lab-ILI). GP-ILI consultations increased from low levels (<1 per GP per day) in week 25 and peaked in epidemiological week 30 (26 July to 1 August 2009), the peak being more pronounced in primary than secondary school-aged children. The proportion of samples positive for pdmH1N1 (Lab-ILI) increased more rapidly than GP-ILI consultation rates, and reached a plateau by week 27 before declining in week 38. The composite GP*Lab-ILI indicator accentuates the epidemic curves, giving a slightly sharper peak for both age groups (Figure 1C).

### School-based monitoring results

**Figure 2:**
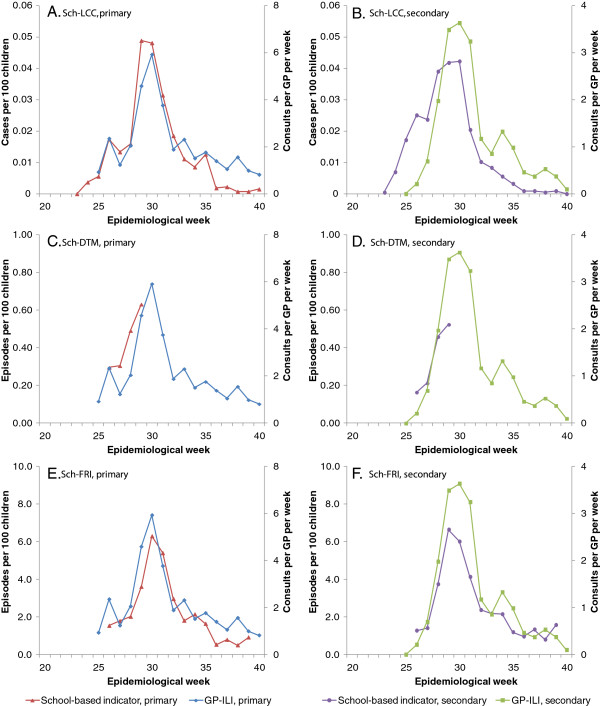
**Comparison of school-based indicators of epidemic activity with GP-ILI activity expressed as consults per GP per week. A** and **B**) Sch-LCC: Notifications of laboratory confirmed pdmH1N1; **C** and **D**) Sch-DTM: Daily temperature monitoring system; **E** and **F**) Sch-FRI: School-based FRI reporting, in primary and secondary schools respectively. Red and purple lines denote data from school-based indicators, while blue and green lines give the GP-ILI activity for primary and secondary schools respectively.

Figure 2 compares the epidemic curves from the various school-based indicators with the GP-ILI data from Figure 1A. Sch-LCC notifications (Figures 2A and B) increased and also declined earlier, particularly in the secondary school age group, while the daily temperature monitoring (Sch-DTM, Figures 2C and D) and FRI reporting (Sch-FRI, Figures 2E and F) tracked the GP-ILI data more closely. Peak incidence between the GP-ILI, Sch-LCC and Sch-FRI indicators were within a week of each other. However, Table 2 shows that by the time GP-ILI incidence peaked in week 30, 63% and 79% of Sch-LCC notifications in primary and secondary school-age children had occurred, compared to about half the total cumulative incidence in GP-ILI (50% and 52%), GP*Lab-ILI (49% and 52%) and Sch-FRI (48% and 53%).

**Table 2:** Comparison of school-based with clinic-based indicators

**Summary measure**	**School type**	**Clinic-based indicators†**		**School-based indicators, per 100 school children‡**		
		**GP-ILI**	GP* Lab-ILI	**Sch-LCC**	**Sch-FRI**	**Sch-FRI-adj**
Cumulative incidence in entire period	Pri	34	21	0.24	32	25
	Sec	19	13	0.25	36	25
Cumulative incidence up to week 30	Pri	17	10	0.15	15	13
	Sec	10	6.9	0.20	19	15
Fraction of cumulative incidence occurring up to week 30	Pri	50%	49%	63%	48%	51%
	Sec	52%	52%	79%	53%	61%

All data has been rounded off to 2 significant figures.

†Incidence data for GP-ILI is expressed as the number of ILI consults per GP per week, while that for GP*Lab-ILI is the number of ILI consults per GP per week attributable to pdmH1N1, as derived by multiplying GP-ILI with the weekly proportion of ILI samples positive for pdmH1N1.

‡Sch-LCC is based on notifications of laboratory confirmed cases of pdmH1N1 by all schools, Sch-FRI is self-reported febrile respiratory illness in the 6 sentinel schools, and Sch-FRI-adj is Sch-FRI adjusted to remove possible contribution from non-pdmH1N1 causes using FRI incidence from week 38 as a proxy for baseline incidence.

Table 2 also gives the cumulative incidence for Sch-LCC and Sch-FRI over the epidemic. There were only 0.24 and 0.25 laboratory confirmed cases (Sch-LCC) for every 100 primary and secondary school-age children respectively. Since our estimate of the proportion infected during the epidemic from serological data was 41.8% (95% CI: 30.2% to 55.9%) in primary and 43.2% (95% CI: 28.2% to 60.8%) in secondary school-age children, this suggests that less than 1% of infections were notified. However, after adjustment to remove the contribution of non-pdmH1N1 causes of FRI (Sch-FRI-adj), there were 25 FRI episodes per 100 children in both primary and secondary school-age children, with the ratio of FRI to estimated infections being 0.60 and 0.57 respectively. The daily temperature monitoring system (Sch-DTM) was curtailed mid-epidemic, but we can compare the incidence rates for Sch-DTM and Sch-FRI in week 29. While there were 3.6 and 6.6 FRIs reported per 100 primary and secondary school-age children in that week, the Sch-DTM system detected only 0.63 and 0.52 fevers per 100 children, giving a rate ratio for Sch-DTM to Sch-FRI of 0.175 (95% CI: 0.150 to 0.209) and 0.079 (95% CI: 0.070 to 0.090) respectively. Using cumulative FRI rates from week 26 to 29 when both systems were active gives similar rate ratios of 0.193 (95% CI: 0.174 to 0.215) and 0.104 (95% CI: 0.096 to 0.114). The amount of febrile illness detected by Sch-DTM was hence from less than a tenth to about one fifth of that detected by the Sch-FRI system.

**Figure 3:**
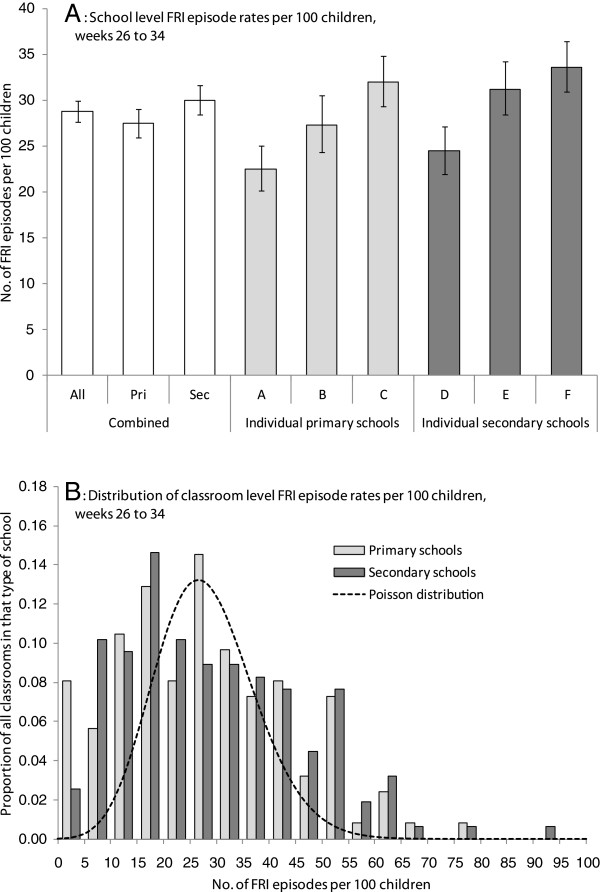
**Distribution of Sch-FRI episodes by schools and classrooms from weeks 26 to 34. A**) School level rates of FRI episodes per 100 children; error bars denote 95% confidence intervals from a Poisson distribution. **B**) Distribution of classroom level rates of FRI episodes per 100 children for 124 primary and 157 secondary school classrooms, in light and dark grey respectively. Dashed line gives the expected distribution based on the combined average of 9.2 FRI episodes per classroom.

**Figure 4:**
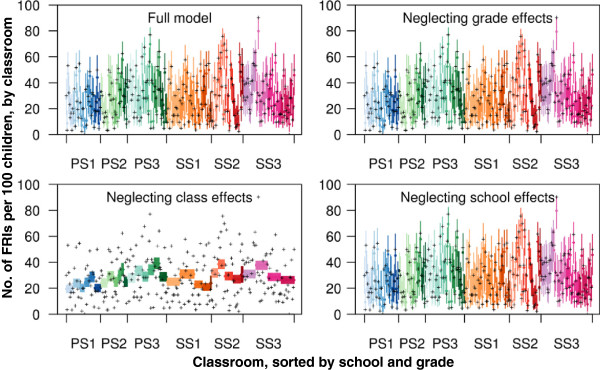
**Modeled and empirical within classroom FRI attack rates within schools for four different hierarchical models.** Classes were sorted first by schools (PS1–3 are primary schools, SS1–3 are secondary schools, indicated by families of colors), then by age group of the class (indicated by different shades of color) and then randomly within age groups. Point estimates (colored dots) are posterior means, while colored lines indicate 95% uncertainty intervals. Empirical proportions are indicated by crosses. The full model contains age, class and school effects; the other models ignore one of these three effects.

Figure 3 elaborates on the Sch-FRI reporting rate from weeks 26 to 34, when data from all 6 participating schools was available. The variation in rates of school level FRI episodes was small with overlapping confidence intervals (Figure 3A), with the mean number of FRI episodes reported for all schools being 28.8 per 100 children (95% CI: 27.7 to 29.9), or about 9.2 per classroom. However, there was substantial variation in classroom level FRI rates, with the distribution being fairly similar between primary and secondary schools, and yet deviating substantially from what would be expected from a Poisson distribution based on the mean number of FRIs reported per classroom (Figure 3B). Figure 4 shows the hierarchical models of FRI rates within classrooms. Deviance information criteria (DIC) for the full model, and the models neglecting age (as represented by class grade) and school effects, all fall within 0.62 of each other. Excluding classroom level effects, however, leads to a highly significant reduction in goodness of fit with a rise in the DIC of 800. This suggests that little of the empirical variability between FRI rates can be explained by school or age group effects, but rather must be explained by heterogeneities at the classroom level.

## Discussion

The incidence and risk factors for pdmH1N1 during the 2009 pandemic were well studied in Singapore [18,20-22]. In this work, cross-sectional serologic surveys suggest that about 40% of school-aged children were infected in the initial wave of pdmH1N1 in Singapore compared with 17% of adults [17]. These results corroborate the importance of school-aged children in influenza transmission, and the potential of schools as a source of influenza sentinel surveillance data, particularly since epidemics in school-aged children tend to lead influenza activity in older age groups [23].

Comparison of the epidemic curves suggests that, while illness episodes from Sch-FRI reporting closely tracked those from GP ILI surveillance, laboratory confirmed pdmH1N1 cases (Sch-LCC) detected more of the earlier infections in the epidemic. This is expected since pdmH1N1 notifications were based on the prevailing national testing and reporting regime. At the start of the local epidemic from late May through June 2009, all individuals suspected of having pdmH1N1 infection were comprehensively tested as part of the Ministry of Health protocol during the containment phase of the epidemic, with this requirement abolished when the national response transitioned to mitigation phase in early July 2009 (in week 27) [13]. Moreover, we noticed that the distribution of pdmH1N1 notifications cases was skewed, with a few schools contributing most of the cases (data not shown) – this may be due to higher testing and reporting rates in schools with earlier outbreaks; other authors have likewise observed how cases linked to outbreaks can be over-represented in laboratory confirmed infections [24].

For the same reasons, we found when comparing incidence rates between the systems that laboratory confirmed pdmH1N1 cases (Sch-LCC) likely detected less than 1% of all estimated infections. Since our clinic-based ILI data started only from week 25, we are unable to estimate the fraction of infections detected by laboratory confirmed cases notified earlier during the epidemic, which may have been substantially higher. However, we note that studies from other developed countries which attempt to estimate infections either by symptoms or serology likewise suggest that only a small proportion of infections are confirmed [25-28]. Based on the comparison with Sch-FRI, temperature monitoring twice a day (Sch-DTM) may also only have identified less than one fifth of febrile respiratory illness episodes, and by extrapolation a smaller fraction of infections. Many influenza infections never result in fever [29,30], and those who do become febrile may not have a fever at the time of monitoring, may refrain from attending school in the first place, may take antipyretics, or may have an elevated temperature that nevertheless falls below the defined threshold; any of these circumstances would result in cases not being identified by the monitoring system. On the other hand, our novel teacher led febrile respiratory illness reporting system (Sch-FRI) covering six schools distributed across the country obtained incidence rates consistent with those observed in some school-based outbreaks where syndromic case definitions of self-reported fever and respiratory symptoms were also used [31-33]. While data specific to pediatric populations is lacking, other studies show that symptoms occur in two thirds to three quarters, and febrile illness in about half of serologically detected infections [18,29,34]. Since our ratio of illness episodes (Sch-FRI-adj) to infections was around 0.6, we suggest that self-reported FRI had detected a substantial proportion of symptomatic infections, and hence may be sufficiently sensitive as a means of detecting clusters of transmission in contrast to the other two indicators (Sch-LCC and Sch-DTM) evaluated, which may be limited in their sensitivity for triggering investigations and interventions.

A surveillance system built upon a small group of schools, as in the Sch-FRI system described here, would not allow central educational authorities to instigate responsive school closures in schools which are not enrolled in the network. However, self-reported ILI has been used successfully to investigate school-based outbreaks [9,33]; others have also used self-reported ILI to assess the burden of pdmH1N1 in the community and the proportions which seek care [24,35]. We believe that the school-based FRI reporting we describe offers some advantages over clinic-based ILI reporting: (i) it can be rapidly implemented in a centralized educational system, as in Singapore, (ii) it is not dependent on health-seeking behavior and can potentially work in areas with poor primary care coverage, (iii) it has clear denominators of the population at risk, and (iv) it does not require additional laboratory testing or serological studies. On the other hand, such systems face several challenges, including how to monitor epidemics during school holidays, the representativeness of participating schools (particularly in rural areas where transmission may be less uniform than in highly urbanized Singapore), mitigating the burden of data collection, and integrating such surveillance with data on adults and pre-school children. However, in spite of these limitations, such a system can be a useful adjunct to other more established clinic-based systems, since it is not dependent on primary care coverage or health-seeking behavior, and allows estimates of infection rates following adequate adjustment for the contribution of other causes of febrile respiratory illness and the proportion of infections that do not present with fever. Our analysis of the variation in FRI rates also suggests that FRI reporting has some potential for identifying localized transmission. We noted a wide difference in FRI rates by classrooms, with more than 10-fold difference in rates between the 5^th^ and 95^th^ percentile (5 vs 58 episodes per 100 children). In spite of this, FRI rates aggregated at the level of schools were relatively similar. We suggest that this apparent disconnect could be explained if we consider influenza incidence at school level as an aggregate of semi-independent self-sustaining clusters of transmission at the class-room level, which produces a wide range of cluster sizes distributed around an inherent mean. When the schools are a sufficiently large collection of classrooms (as was the case in our study, where the 6 schools had between 33 to 63 classrooms, with a median class size of 31 students and inter-quartile range from 29 to 39), then the school level FRI rate reflects the average size of a transmission cluster in the classroom setting. Cauchemez et al. have demonstrated that, within the school environment, classroom level transmission dominates [4], and our study adds to the emerging evidence that this is indeed the case. Notably, in the Singapore school system, students mostly interact within the same class, with most classes conducted within the same room throughout the day for lessons, and this may have accentuated the effect. Additional studies will be needed to clarify the pattern of influenza transmission within schools, as this will have substantial implications on control measures, since execution of closures and interventions at classroom level, if effective, would be far less disruptive than equivalent measures at the level of entire schools or even all schools within geographic areas. However, if there is intent to intervene using such data, then febrile respiratory illness may have to be monitored in real-time and followed-up by confirmatory testing of students identified, which may be logistically challenging since some students would be absent from school at the time of their illness; this may also be expensive if implemented at a national level. There may also be issues with variations in data quality if deployed on a wider scale or for longer periods, and as such FRI reporting may function best either when used for short periods such as for detecting transmission clusters during severe epidemics, or in sentinel schools with dedicated support staff to ensure that it is properly collected when used for long-term surveillance of influenza activity. Finally, modeling studies should be attempted to suggest appropriate triggers for interventions (such as a certain number of FRI episodes within a particular time frame), and the potential effect of any interventions on reducing influenza transmission.

Limitations of our study include the fact that the different indicators were collected over different time periods, especially for daily temperature monitoring which was only available for a limited period according to the Ministry of Education mandate. Furthermore, it is not possible on the basis of our analysis alone to determine which of the different systems most accurately reflected the true timing of the epidemic, especially in view of data availability, reporting lags and day of week effects which limited our resolution to weekly incidence and may have introduced bias from one or more datasets. Moreover, missing onset dates from about a quarter of the reported FRI episodes may have biased the epidemic curve for this dataset. Ideally, we would also have wanted information on all presentations of acute respiratory illness, with additional information on the recorded temperature, and not just FRI; however, this would have increased the complexity of data collection, and we therefore adopted the compromise of using just febrile respiratory illness as our case definition for identifying possible pdmH1N1 infections. In addition, we had inadequate data on the baseline incidence of FRI, and this may have biased our estimation on the contribution of non-pdmH1N1 causes to FRI. Notably, one study from the US suggests a substantially higher baseline incidence of self-reported febrile respiratory illness in children (ages < 18 yrs), although the case definitions used in that study were slightly less specific [36]. Some routine collection of baseline FRI incidence may hence be necessary to aid interpretation of data from FRI monitoring systems during epidemics and pandemics. Also, the serological survey that was used for comparison had a post-epidemic sample which was taken from 1 October 2009 to 2 June 2010. Subsequent smaller waves of H1N1 2009 within that period [37] and waning of initial antibody levels [38,39] might have affected the results. It must also be noted that the data used to adjust for the sensitivity of the HI assay was based on results from adults [19], since we did not have data specific to younger populations. However, we note that the estimates of infection rates in our study are fairly similar to other serologic surveys of corresponding age groups in other countries following a single epidemic wave [1,26,28,40-42]. Finally, our study lacked the necessary data such as differences in interventions or time lag between symptom onset and sickness absenteeism to explain our intriguing finding on the large variation in clinical attack rates at classroom level.

## Conclusions

Our study shows that notifications of laboratory confirmed influenza (Sch-LCC) and school-wide daily monitoring of temperature (Sch-DTM) were unlikely to have detected a sufficient fraction of influenza cases to allow effective school-level interventions. On the other hand, we believe that school-based FRI reporting by teachers at sentinel schools can be reasonably accurate in monitoring influenza epidemics and may be particularly useful in resource poor settings as the costs are minimal. Our FRI data also revealed wide-spread variation at the level of classrooms (but not schools), and corroborates other evidence that classroom-based transmission may dominate. FRI reporting in schools could hence be a potential method for detecting transmission clusters in future pandemics or even severe seasonal influenza epidemics when interventions such as school closures and targeted administration of antiviral treatment and prophylaxis [43] are being considered.

## Competing interests

PAT received research support from Baxter, GSK, Sanofi-Pasteur, ADAMAS and honoraria from Novartis, MSD and Astra-Zeneca unrelated to this study.

## Authors’ contributions

ARC, VJL and MICC conceived the study, and SMS, PAT and DLMG were involved in refining the design. SES implemented the study and performed field work. JLC, VTKC, MCP, NWST and SKS provided samples for serological analyses. CL, RTPL and IGB organized and provided laboratory assays for influenza serology. LWA, WYL and LGG provided survey data for analysis. ARC and MICC performed the analyses. YCC, KCT, YSL provided additional inputs on the interpretation of the findings. SES and MICC drafted the initial manuscript. All authors read and approved the final manuscript.

## Pre-publication history

The pre-publication history for this paper can be accessed here:

http://www.biomedcentral.com/1471-2334/12/336/prepub

## Supplementary Material

Additional file 1 **Technical appendix and supplementary data **[18,19,44]**.** Click here for file
