# Supplementary material for: Teacher led school-based surveillance can allow accurate tracking of emerging infectious diseases - evidence from serial cross-sectional surveys of febrile respiratory illness during the H1N1 2009 influenza pandemic in Singapore
Source: BMC Infect Dis. 2012 Dec 4;12:336. doi: 10.1186/1471-2334-12-336 (PMC3544582; doi:10.1186/1471-2334-12-336)
Supplement: Additional file 1 — Technical appendix and supplementary data [18,19,44]. [file 1471-2334-12-336-S1.docx]

Technical appendix and supplementary data

Serological investigations

We estimated the seroprevalence of antibodies to pdmH1N1 strain A/California/7/2009 in residual sera obtained from the two national pediatric hospitals, National University Hospital (NUH) and Kandang Kerbau Women's and Children's Hospital (KKH). These sera included 975 samples from the Singapore National Pediatric Seroprevalence Survey, a large seroprevalence study started before the pandemic in August 2008 and completed in July 2010 [44]. To increase the sample size for age-stratified analysis, 400 additional samples of residual sera from children attending KKH were collected in May 2010. Samples were tested by hemagglutination inhibition (HI) assays using A/California/7/2009 H1N1 at the World Health Organization Collaborating Centre for Reference and Research on Influenza in Melbourne, Australia, as previously described [18].

We then estimated the cumulative incidence of pdmH1N1 infection over the time period of interest by taking the difference in antibody prevalence at HI titers of ≥40 between pre-epidemic (Pre-SS, *s_1_*) and post-epidemic samples (Post-SS, *s_2_*), and accounting for the fraction of infections in those with pre-existing antibodies at titers of ≥40, and adjusting this upwards for the imperfect sensitivity of the HI assay (since our previous study found that only 77.8% achieved titers of ≥40 following infection by the same HI assay) [19], as follows:

$$S\text{ }=\text{ }\left(s_{2}-s_{1}\right)/\left[\left(1\text{ }-s_{1}\right)\times\left.0.778\right)\right]$$

The table below provides the actual data used in our estimates and the results.

Appendix Table 1. Serological data and estimated proportions infected by age

| **Age group** | **% with HI titers ≥40** | | **Estimated proportion infected:** ***S^A,B,C,D^*** |
| --- | --- | --- | --- |
|  | **Pre-epidemic sera, % (n/N)†** | **Post-epidemic sera, % (n/N)†** |  |
| A: 7 – 12 yrs | 0.8% (3/381) | 33.1% (41/124) | 41.8% |
| B: 13 – 16 yrs | 10.6% (34/321) | 40.6% (39/96) | 43.2% |
| C: 0 – 4 yrs | 0.0% (0/80) | 25.0% (34/136) | 32.1% |
| D: 5 – 6 yrs | 0.0% (0/64) | 23.4% (11/47) | 30.1% |

†n is the number with HI titers ≥40 while N is the total number of sera tested.

Age groups A and B are used as proxies for infections in secondary and primary school children respectively. Age groups C and D give the data for pre-school and pre-primary children; these were not used in our study but are presented above and mentioned in the discussion for comparison with school-going ages.

Quantifying relative importance of school, age group, and classroom on variability of FRI rates

To quantify the relative importance of three sources of variability (school, age group, and classroom) on classroom FRI rates, we developed a hierarchical model in which the logit of the per capita probability of having an FRI was governed by school, age and classroom effects. The full model for data and parameters was:

$$\begin{matrix} \log\frac{p_{i}}{1-p_{i}}=a+b_{G_{i}}+c_{S_{i}}+d_{i} \\ F_{i}\sim\text{Bin}\left(n_{i},p_{i}\right) \\ b_{i}\sim N\left(0,\sigma_{b}^{2}\right) \\ c_{i}\sim N\left(0,\sigma_{c}^{2}\right) \\ d_{i}\sim N\left(0,\sigma_{d}^{2}\right) \\ a\sim U\left(-1000,1000\right) \\ \sigma_{b}\sim U\left(0,1000\right) \\ \sigma_{c}\sim U\left(0,1000\right) \\ \sigma_{d}\sim U\left(0,1000\right) \end{matrix}$$

where

- *p_i_* is the per-capita FRI probability for classroom *i*;
- *F_i_* is the number of FRIs in classroom *i*;
- *n_i_* is the enrolment of classroom *i*;
- *G_i_* is the grade (i.e. age group level) of classroom *i*;
- *S_i_* is the school containing classroom *i*.

The restricted, more parsimonious models excluded terms *b*, *c*, and *d* and relevant prior distributions from the equations above.

The results were plotted in Figure 4, using families of colors to represent the schools, and shades of these colors to indicate the age group of the class. The families of colors used were derived with the help of the software, Colorbrewer (http://www.personal.psu.edu/cab38/ColorBrewer/ColorBrewer.html).
